# Targeting ubiquitin specific proteases (USPs) in cancer immunotherapy: from basic research to preclinical application

**DOI:** 10.1186/s13046-023-02805-y

**Published:** 2023-09-01

**Authors:** Hongli Gao, Jianqiao Yin, Ce Ji, Xiaopeng Yu, Jinqi Xue, Xin Guan, Shuang Zhang, Xun Liu, Fei Xing

**Affiliations:** 1 Department of Oncology, Shengjing Hospital of China Medical University, Shenyang, 110004 China; 2 Department of General Surgery, Shengjing Hospital of China Medical University, Shenyang, 110004 China; 3 Department of Gastroenterology, Shengjing Hospital of China Medical University, Shenyang, 110004 China

**Keywords:** Ubiquitin-specific proteases (USPs), Cancer, Immunotherapy, USP inhibitors

## Abstract

Tumors have evolved in various mechanisms to evade the immune system, hindering the antitumor immune response and facilitating tumor progression. Immunotherapy has become a potential treatment strategy specific to different cancer types by utilizing multifarious molecular mechanisms to enhance the immune response against tumors. Among these mechanisms, the ubiquitin–proteasome system (UPS) is a significant non-lysosomal pathway specific to protein degradation, regulated by deubiquitinating enzymes (DUBs) that counterbalance ubiquitin signaling. Ubiquitin-specific proteases (USPs), the largest DUB family with the strongest variety, play critical roles in modulating immune cell function, regulating immune response, and participating in antigen processing and presentation during tumor progression. According to recent studies, the expressions of some USP family members in tumor cells are involved in tumor immune escape and immune microenvironment. This review explores the potential of targeting USPs as a new approach for cancer immunotherapy, highlighting recent basic and preclinical studies investigating the applications of USP inhibitors. By providing insights into the structure and function of USPs in cancer immunity, this review aims at assisting in developing new therapeutic approaches for enhancing the immunotherapy efficacy.

## Background

Tumors are considered to have particular growth, invasion, and metastatic properties. Recently, scientists' focus has been drawn to cancers' capacity to evade immune system and avoid immune detection, which would trigger an antitumor immune response to create a favorable environment for tumor growth and survival [1, 2]. One of the key mechanisms of immune evasion is production of immunosuppressive mediators and cytokines. These immunosuppressive factors can inhibit the activation and proliferation of immune cells, thereby limiting the immune response against tumors [3]. Additionally, tumors may also downregulate major histocompatibility complex (MHC) molecule expression, which essentially helps to present tumor antigens to T cells, thereby reducing their ability to transmit antigens to T lymphocytes [4]. Furthermore, tumors can recruit special immunoregulatory cells, like regulatory T cells (Tregs) and myeloid-derived suppressor cells (MDSCs), to their specific microenvironment. These cells contribute to the immunosuppressive milieu by releasing factors that inhibit the activity of immune cells. Tregs are capable of directly inhibiting the activity exhibited by effector T cells, while MDSCs can prevent natural killer (NK) cells, dendritic cells (DCs) and neutrophils from being activated [5–7]. The recruitment of immunoregulatory cells can also promote tumor angiogenesis and metastasis, further facilitating tumor growth and progression [8]. Therefore, tumors possess various mechanisms to evade the immune system, strategies to overcome these mechanisms and enhance the immune response against tumors may represent promising avenues for cancer treatment.

Immunotherapy has been developing to be a largely potential treatment strategy specific to different cancer types recently. Unlike traditional chemotherapy, which targets both cancerous and healthy cells, immunotherapy aims to harness the patients’ own immune system for selectively removing tumor cells [9–11]. For example, immune checkpoint inhibitors (ICIs) block the interaction between immune checkpoint molecules and their ligands, thus preventing the exhaustion of T cells and enhancing their antitumor activity [12]. CD274, also known as Programmed Cell Death 1 Ligand 1 (PD-L1) and B7 homolog 1 (B7-H1), is a critical immune checkpoint that effectively regulates the immune response. It is a ligand for the immune inhibitory receptor CD279, also known as PD-1, which shows expression on activated T cells [13, 14]. Due to the binding of PD-1 to PD-L1, T cell activation and function are inhibited [15]. Various antibody-based drugs that target the PD-1/PD-L1 interaction are developed as a potential strategy for restoring the effector response of cytotoxic T cells to tumors and enhancing the antitumor immune response [16, 17]. Besides, adoptive cell transfer could also involve the infusion of ex vivo expanded T cells to specifically recognize and kill tumor cells [18]. Additionally, cancer vaccines can stimulate the immune system to recognize and attack tumor cells [19, 20]. Immunotherapy drugs, such as pembrolizumab and nivolumab, have been shown to effectively treat various solid tumors, including non-small cell lung cancer (NSCLC), bladder cancer, melanoma, and renal cell carcinoma (RCC) [21–23]. However, it is important to note that not all cancers respond equally well to immunotherapy. Resistance to treatment can develop in up to 60% of patients who initially respond to immunotherapy, and even advanced therapies like ICIs can demonstrate limited efficacy [24]. Therefore, developing new therapeutic modalities capable of strengthening immunotherapy efficacy is the most significant. Combining immunotherapy with other treatment options, such as targeted therapy, radiation therapy or chemotherapy, may represent a more promising strategy to overcome resistance and improve therapeutic rates.

The controlled regulation of protein turnover can essentially maintain stable cell structure and function. Approximately 30% of proteins that are newly synthesized in mammalian cells have a short half-life of less than 10 min, and must be rapidly degraded [25]. To achieve such high protein turnover rate, cells have evolved a specialized system for the selective and controlled unwanted protein degradation. This system includes the ubiquitin–proteasome pathway, lysosomal degradation and autophagy [26, 27]. Among these, ubiquitin–proteasome system (UPS) is a typical non-lysosomal protein degradation pathway. Ubiquitination serves as a crucial post-translational modification process responsible for regulating protein activation/inactivation, signal transduction, gene regulation, and DNA repair [28, 29]. This ubiquitination process involves ubiquitin (Ub) molecules being covalently attached to substrate proteins via iso-peptide bonds under the catalysis of the E1-E2-E3 ligase cascade [30]. The ubiquitination process is reversible and called deubiquitination [31]. During deubiquitination, deubiquitinating enzymes (DUBs) play a crucial role in mediating how removing covalently attached ubiquitin moieties from substrate proteins [32]. DUBs reverse the Ub attachment for counterbalancing ubiquitination signaling, meanwhile promoting Ub molecule recycling [33]. Stable regulation of DUBs can necessarily assist in controlling the cell biology and physiology, while DUB turnover defects can contribute to disease development, like neurodegenerative, autoimmune and inflammatory disorders, infections, and cancers [34, 35]. To date, there are over 100 DUBs identified in humans, which fall into 6 families considering structure and function, namely ubiquitin-specific proteases (USPs), ubiquitin C-terminal hydrolases, ovarian tumor proteases, Machado-Joseph disease protein proteases, the motif interacting with novel DUB family containing ubiquitin, and Zinc Finger USP [36, 37]. USPs making up around 60% of all DUBs, are the largest and most varied DUB family [38]. More and more studies have demonstrated that USPs can regulate the efficacy of immunotherapy through modulating immune cell function and immune response in tumor microenvironment (TME) [39–42]. The expressions of some USPs in tumor cells also has been revealed to be related to tumor immune escape and to mediate the drug resistance [43].

Hence, this review will focus on exploring the specific mechanisms by which USPs participate in cancer immunity, including their involvement in antigen processing and presentation, immune cell activation and regulation, and the modulation of immune checkpoint pathways, etc. We will also discuss recent basic and preclinical studies investigating the applications of USP inhibitors on cancer immunotherapy, and demonstrating the challenges and opportunities associated with this emerging field. This review is the first to provide a comprehensive summary of the current state of USPs and cancer immunity, highlighting its significant interest and relevance, serving as a valuable resource for researchers and clinicians looking to stay up-to-date on the latest developments in USP members and cancer immunotherapy.

## USP7

### Structure of USP7

Among the almost 100 deubiquitinating enzymes, USP7 is the most widely studied in various researches [35, 44]. USP7, a 135 kDa cysteine protease comprised of 1102 amino acids includes 1 catalytic core domain, 5 C-terminal ubiquitin-like domains (UBL1-5) and 1 N-terminal Tumor necrosis factor Receptor-Associated Factor (TRAF)-like domain [45–47]. The catalytic domain of USP7 (residues 208–560) lies between TRAF-like domain and UBL domain, and is flanked by an N-terminal domain [48]. The catalytic core domain has a major function of cleaving the iso-peptide bond between the Ub and the substrate protein, and consists of the amino acid residues Cys223, His464, and Asp481, to cooperatively participate in substrate deubiquitination [49–51]. The UBL domains 1, 2, and 3 participate in the binding interactions with various proteins, while UBL domains 4 and 5 crucially impact the complete deubiquitinated activity [52]. Among all USP family members, only USP7 has the special TRAF-like domain (residues 53–205), which is needed for the recognition of substrates [52]. After recognizing and interacting with a ubiquitinated substrate by TRAF-like domain, the catalytic activity will spatially rearrange through conformational change. Additionally, the C-terminal residues can essentially assist in the activation of the catalytic activity, whereas the N terminus assists in the nuclear localization [48]. These regulatory function of the catalytic domains, UBL domains, with the unique TRAF-like domain, together contributes to the substrate recognition and specificity of USP7.

### Immunoregulatory function of USP7 in cancers

**Fig. 1:**
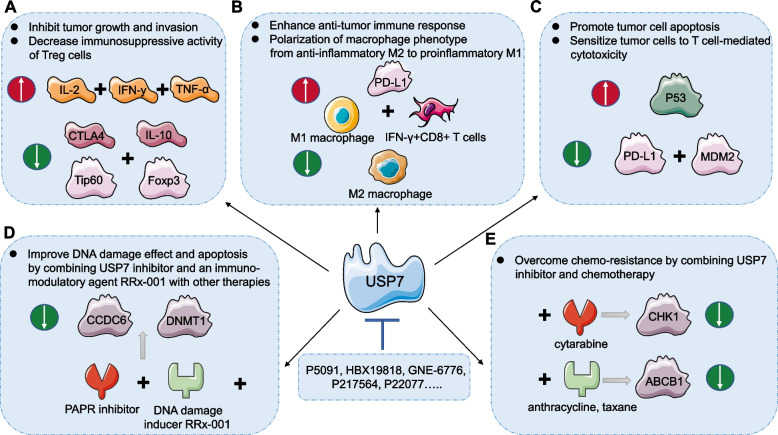
The effect by targeting inhibition of USP7 on anti-tumor immune response: **A** Inhibit tumor growth and invasion, and decrease immunosuppressive activity of Treg cells by suppressing the formation of Tip60-mediated Foxp3 dimers that bind to CTLA4 and IL-10 genes, while simultaneously upregulating the expression of IL-2, IFN-γ and TNF‐α genes. **B** Promote M2 macrophages to polarize to M1 macrophages, increase tumor infiltration of IFN-γ + CD8 + T cells, also upregulate PD-L1 expression. **C** Inhibit PD-L1 expression and promote p53-dependent apoptosis in tumor cells via p53/MDM2 pathway. **D** Identify CCDC6 and DNMT1 degradation to enhance the effect of combination PARP-inhibitor and DNA damage inducer RRx-001. **E** Overcome chemoresistance by combing USP7 inhibitor and cytarabine through downregulating CHK1 protein expression in leukemic, also by combing USP7 inhibitor with anthracycline and taxane through downregulating ABCB1 resistant protein expression in triple negative breast cancer

Scientists have made significant advances in understanding how USP7 modulates cancer patients’ immune response in recent years. Within the TME, the highly immunosuppressive Forkhead box protein P3 (Foxp3) + Tregs can limit the antitumor responses presented by effective T cells [53, 54]. It has been demonstrated that higher levels of USP7 facilitate the growth of tumors by modifying the immunosuppressive properties of Foxp3 + Treg cells [55–57]. In the ex vivo T_eff_ suppression test by van Loosdregt et al., USP7 could interact with Foxp3 in Tregs; and USP7 knockdown hindered Tregs’ functions [57]. USP7 critically also impacts Treg cell survival through deubiquitinating and stabilizing the histone acetyltransferase tat-Interactive protein (Tip60). Targeting USP7 may disrupt Foxp3 dimer formation mediated by Tip60, leading to decreased activation of cytotoxic T-lymphocyte-associated protein 4 (CTLA4) and IL-10, and increased IL-2 and IFN-γ expression (Fig. 1A) [57].

Tumor-associated macrophages (TAMs) is a type of infiltrating inflammatory cells differentiating into M1 and M2 phenotype depending on the signals present within TME. The M1 phenotype is characterized by its ability to suppress tumor growth and promote an anti-tumor immune response. In contrast, the M2 phenotype is associated with tumor promotion and immune suppression [58]. USP7 is an indispensable gene that affects M1 and M2 macrophages' homeostasis. When USP7 is specifically suppressed, M2 macrophages change in terms of the phenotype and functions, leading to increased proliferation of differentiation CD8 + T cell cluster in vitro [59]. As for Lewis lung carcinoma mice, treatment with the USP7 inhibitor weakened the tumor growth as well as increased the infiltration of M1 macrophages and CD8 + T cells expressing IFN-γ through the mechanism underlying activating the p38 mitogen-activated protein kinase (MAPK) pathway. Notably, such therapeutic effect was weakened by TAMs depletion [59] (Fig. 1B).

In several types of cancer, such as gastric tumors, overexpression of USP7 has been observed, and its expression levels exhibit a positive relevance to PD-L1 expression [60]. Hence USP7 may assist in stabilizing PD-L1 protein levels, potentially playing a role in tumor immune evasion. However, previous Dai’s study found that USP7 inhibition actually elevated the PD-L1 expression in lung cancer cells (Fig. 1B) [59]. These suggest that the regulatory effect of USP7 on PD-L1 expression is complex and may be context-dependent. Even so, USP7 inhibitors being combined with PD-1 inhibition or PD-L1 inhibition have shown promise in improving antitumor responses [61]. Along with its immunomodulatory capabilities, USP7 also controls the activation regarding essential transcription factors to regulate immune process like tumor suppressor p53. On one hand, USP7 was discovered to directly deubiquitinate and stabilize p53 to inhibit tumor cell growth and activate apoptosis [62]. On the other hand, USP7 also can bind to and stabilize E3 ubiquitin ligase MDM2, a negative regulator of p53 [63]. During the deubiquitination process of USP7 towards these two proteins, USP7 overexpression causes MDM2 to be deubiquitinated more thoroughly than p53, accelerating the degradation of p53. Thus, inhibition of USP7 results in MDM2 degradation, the MDM2 levels available are no longer sufficient for ubiquitination, which in turn improve p53 stability to enhance anticancer therapeutic efficacy shown both in vitro and in vivo investigations (Fig. 1C) [64]. APG-115, a type of MDM2 antagonist functions as a pharmacological p53 activator. For mutant p53 solid tumor patients, a phase 1b clinical trial (NCT03611868) is now being conducted to assess the safety and effectiveness of APG-115 combined anti-PD-1 pembrolizumab therapy [65]. As previously mentioned, USP7 inhibitors could enhance in tumor cells’ PD-L1 levels, potentially enhancing the anti-PD-1 therapy efficacy. Therefore, it may be highly effective to boost cancer immunotherapy by the co-treatment of USP7 inhibition plus MDM2 blockage and anti-PD-1 therapy [65] (Fig. 1C).

### The developments and applications of USP7 inhibitors in cancers

In chemical biology studies, USP7 is validated as a target of tumor survival [66]. USP7 is overexpressed in many cancer types, such as ovarian cancer, breast cancer (BC), multiple myeloma, hepatocellular carcinoma, glioblastoma, colorectal cancer (CRC), neuroblastoma, squamous cell carcinoma, and lung cancer [66–73]. The USP7 overexpression often represents weaker prognosis in neuroblastoma, lung cancer, and CRC [67–69, 71]. To inhibit the overexpression of USP7 in cancers, the first significant breakthrough in USP7 inhibitor development was made by Chauhan et al., who demonstrated that P5091 as a USP7 inhibitor, restricted multiple myeloma growth in MM1.S xenograft models [66]. With the deepening of research in recent years, more USP7 inhibitors have been developed and can be grouped into five groups according to their primary scaffolds: acridine, substituted thiophene, indeno [1,2-b] pyrazine, quinazolin-4-one, and the derivatives of 2-amino 4-ethylpyridine [74]. These inhibitors function in two distinct manners: covalent or non-covalent interactions with the target. By creating covalent bonds and preventing the interaction of Ub with the catalytic domain of USP7, the covalent inhibitors primarily target USP7 catalytic domain at Cys223 [75]. The Ub-PLA2 enzyme reporter assay was used to screen of a compound library including P5091, P22077, and P217564 (derivatives of P5091), which are typical covalent inhibitors belonging to substituted thiophene derivatives [76]. When these inhibitors bound to USP7, the thiol ring of Cys223 at active site would strike the C-5 thiophene, while the 2,4-difluorobenzenethiol moiety was separated to create the CeS link [77]. Another covalent inhibitor, HBX19818 is an acridine derivative, and electrospray ionization mass spectrometry (ESI–MS) tests revealed that the S atom of Cys223 at active site might establish a covalent connection with C-9 of HBX19818 [78]. However, the selectivity of the aforementioned covalent compounds is limited because of their high homology with the UBP catalytic domain. Instead, the non-covalent inhibitors have superior selectivity because they primarily interact with the allosteric site close to the catalytic center [75]. GNE-6640 and GNE-6776 are two typical non-covalent inhibitors which can bind with an allosteric location 12 Å away from the catalytic Cys223 residue [79]. The important interactions were hydrogen bonds between the 2-amino group and Asp349 and the 4-hydroxy group of the phenol ring and His403 [80, 81].

On the basis of these researches, more and more studies on applications of USP7 inhibitors in cancer immunity has emerged. When used in combination, an adenovirus-based vaccine and P5091 in a mouse CT26 xenograft model displayed better outcomes than either drug used alone, significantly slowing the growth of CRC tumors [22]. After P5091 therapy, the anti-inflammatory cytokine IL-10 was lowered, yet IFN-γ and TNF-α levels were elevated (Fig. 1A). Along with these modifications, the cytotoxic activity exhibited by CD4 + and CD8 + T cells was improved and Foxp3 levels in CD4 + T cells was downregulated, which suggesting probable Treg suppression [22]. In addition, we introduced Dai’s research to explore the relationship between USP7 and M1/M2 macrophages before [59]. 3 USP7 inhibitors, including P5091, HBX19818, and GNE-6776, were used to show that whereas the M1-associated marker CD86 was unaffected by USP7 inhibition, but the M2-associated marker CD206 presented considerably reduced expression (Fig. 1B) [59]. This may implies that USP7 inhibition promotes TAM polarization into proinflammatory M1 macrophages by specifically suppressing M2 macrophages [59].

As the affinity and DUB selectivity exhibited by P5091 are improved, other USP7 inhibitors are developed, including P22077 and P217564 [82]. Both P5091 and P217564 have been shown to selectively interfere with the immunosuppressive functions by downregulating Foxp3 and Tip60, impairing the suppressive function possessed by Tregs [57, 82]. In mouse models of Treg-dependent tumors, such as E7 + TC1 lung adenocarcinoma and AE.17 mesothelioma, P217564 significantly abrogated tumor growth [57]. More important, USP7 inhibition was capable of potentiating the efficacy exhibited by the adenovirus-based tumor vaccine and the anti-PD-1 monoclonal antibody therapy in mice with TC1 lung tumor [57]. Almac4 as another critical USP7 inhibitor, has been demonstrated to decrease tumor cell membrane PD-L1 levels, to attenuate the interaction between PD-L1 and PD-1, then making GC cells more sensitive to cytotoxicity mediated by T cells [60] (Fig. 1C).

Remarkably, inhibition of USP7 can decrease DNA methyltransferase 1 (DNMT1) activity [83]. RRx-001 as a unique, first-in-class epigenetic and immunomodulator drug also can decrease DNMT1 levels to trigger DNA damage response and apoptosis [84]. In multiple myeloma and several preclinical models, RRx-001 plus P5091 combination leads to synergistic anti-tumor efficacy by strengthen DNA damage response [85]. Moreover, in high-grade urothelial bladder cancer, the CCDC6 degradation caused by P5091 making bladder cancer cells more sensitive to PARP-inhibitor drugs [86]. Therefore, combining PARP-inhibitor drugs with USP7 inhibitor besides the DNA damage inducer RRx-001, contributes to new immunotherapeutic strategy specific to cancer patients (Fig. 1D).

In addition, USP7 helps to facilitate drug resistance development through by maintaining the stability of proteins implicated in particular signaling pathways [87]. One of these proteins is Checkpoint kinase 1 (CHK1), which is involved in the replication fork reset during DNA replication process and makes cells more adaptive to DNA damage induced by cytarabine. The cytarabine plus USP7 inhibitor P22077 combination can work synergistically to promote anti-leukemic action, which assist cancer cells overcome chemoresistance [88]. In BC, combination of the USP7 inhibitor with the trastuzumab weakened tumor growth in the xenografts model from a HER2-positive BC patient [89]. And in triple-negative BC (TNBC), the special BC type that is currently approved for immunotherapy, USP7 inhibitor GNE-6776 successfully caused apoptosis, inhibited metastasis and remarkably elevated the chemo-sensitivity by disrupting the interaction between USP7 and ABCB1 (Fig. 1E) [90]. This effect of USP7 inhibitor may enhance specific immunotherapy of TNBC in future.

## USP22

### Structure of USP22

The open reading frame of USP22 takes charge of encoding a peptide with 525 amino acids, and its molecular weight is about 60 kDa [91]. The USP22 structure can be mainly explained based on its yeast homologue, and ubiquitin carboxyl-terminal hydrolase 8 (Ubp8) [92]. An N-terminal zinc finger together with a C-terminal catalytic domain make up USP22/Ubp8. In contrast to other ubiquitinating enzymes, the zinc-finger ubiquitin-binding domain (ZnF UBP) of USP22/Ubp8 fails to directly bind to Ub. Instead, it binds to several other proteins to form tetrameric deubiquitinase module (DUBm) with tight locking. The DUBm deubiquitinates its target proteins to alter their expression [93]. Among these substrate proteins, cyclin D1, c-Myc, sirtuin 1 (SIRT1), B cell-specific Moloney murine leukemia virus integration site 1 (BMI-1), nuclear factor of activated T cells 2 (NFATC2), and far-upstream element–binding protein 1 (FBP1) have been identified to date [94]. These proteins remarkably impact the cell cycle progression, DNA repair, and other cellular processes that are essential for tumor growth and survival [95].

### Immunoregulatory function of USP22 in cancers

**Fig. 2:**
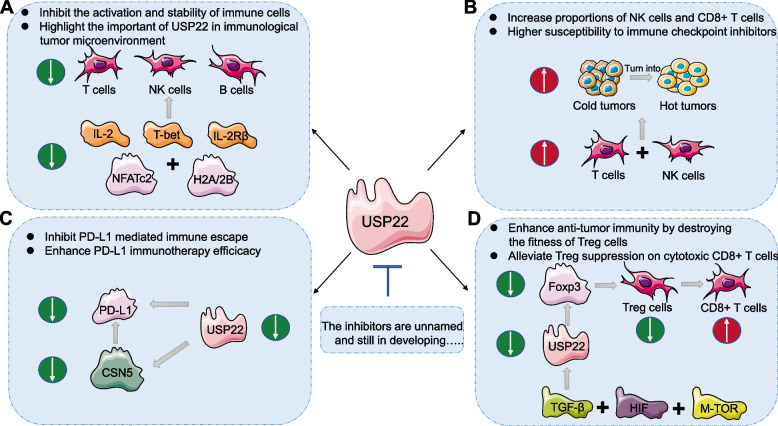
The effect by targeting inhibition of USP22 on anti-tumor immune response: **A** Inhibit T cells, NK cells and primary B cells activation by downregulating the transcriptional activation of T-bet and IL-2Rβ genes through ubiquitination of H2A and H2B. **B** Increase proportions of NK cells and CD8 + T cells in the tumor microenvironment and promote “cold” tumor turn into “hot” tumor which can respond to immunotherapy. **C** Inhibit PD-L1 mediated immune escape and enhance PD-L1 targeted immunotherapy efficacy by directing downregulating PD-L1 de-ubiquitination or through USP22/CSN5/PD-L1 axis. **D** Enhance anti-tumor immunity by decreasing Foxp3 expression to destroy the fitness of Treg cells which regulated by TGF-β, HIF and m-TOR, and alleviate Treg suppression on cytotoxic CD8 + T cells

According to increasing evidence, USP22 appears to display a vital regulatory role in immune system through affecting the growth, development, and phenotypic switching of T cells and B cells. USP22 can deubiquitinate and stabilize NFATC2 to activate T cells and upregulate IL-2 release. USP22 is also required for invariant NK T cell development in early stage [96]. Furthermore, USP22 promotes IL-2 receptor beta (IL-2Rβ) and T-box transcription factor (T-bet) genes activated via H2A deubiquitination [97]. Additionally, USP22 can repair programmed DNA breakage by deubiquitinating H2B-K120 in vivo. The ablation of USP22 could lead to a lack of phosphorylated histone H2AX and damage to classic nonhomologous end joining result from primary B cells [98]. Overall, the significance of USP22 in regulation of immune cells emphasizes its potential as an immunotherapeutic target (Fig. 2A).

In cancers, USP22 is possibly capable of reprogramming the TME and influence the response to immunotherapy. Tumors can be classified into "hot" or "cold" based on whether they are or are not infiltrated by lymphocytes. "Hot" tumor exhibits immunogenicity and is capable of responding to immunotherapy, while "cold" tumor lacks immune cell infiltration and is resistant to immunotherapy [99]. For pancreatic ductal adenocarcinoma (PDAC), targeting USP22 has been shown to enhance the response to immunotherapy and associated with increased proportions of CD8 + T cells and NK cells, which turn “cold” tumor into “hot” (Fig. 2B) [100]. The same results are obtained in liver tumors, when suppressing USP22 increases tumor immunogenicity, encourages T cells infiltration and improves susceptibility to anti-PD-L1 immunotherapy as well as cisplatin-based chemotherapy [101]. When discussing PD-L1, the regulatory role of USP22 towards it is diverse. USP22 has the ability to directly modulate PD-L1 stability via deubiquitination. Knockdown USP22 lowers tumor metastasis dependent of T cells, and enhances NK cells activity, as well as improves anti-PD-1/PD-L1 efficacy [100, 101]. Besides, USP22 is responsible for regulating the PD-L1 protein level by the USP22/CSN5/PD-L1 axis. CSN5 has been discovered as a critical protein that promotes PD-L1 deubiquitination. USP22 deubiquitinates CSN5 polyubiquitin chains and stabilizes CSN5 protein, resulting in enhanced PD-L1 expression [102]. Therefore, USP22 and CSN5 work together to stable PD-L1 expression in cancer cells (Fig. 2C).

As previously noted, Foxp3 + Treg cells can limit effector T cells’ function as well as increase tumor immune evasion. In particular, inhibiting USP22 by CRISPR in Treg cells has been demonstrated to lower Foxp3 protein production as well as reduce tumor growth in different tumor types [103]. Besides, studies have shown that suppression of USP22 in NSCLC can initiate STAT1 signaling then to increase the expression of IFN-γ, a cytokine that critically assists in the activation and proliferation regarding T cells and NK cells [104].

### The developments and applications of USP22 inhibitors in cancers

Unlike USP7, USP22 does not have any small-molecule inhibitor specific to act. In 2021, Morgan et al. published a study that provides a novel alternative strategy for selective target to USP22 [105]. Because isolated USP22 did not exhibit a measurable activity, suggesting that the adapter proteins are necessary for the complex’s DUB activity. Morgan et al. utilized reconstituted human SAGA DUBm, which contains USP22, ATXN7, ATXN7L3, and ENY2, along with fluorogenic Ub-AMC (substrate) to screen cyclic peptides and got six compounds tightly binding to the DUBm [105]. Furthermore, a more important landmark USP22 inhibitor study was published in 2022 [106]. The filtered unnamed compound S02 bound tightly in the catalytic domain pocket of USP22 through side chain–negative residues (Glu and Asp), not the positively charged residues (Arg and Lys). In this study, by regulating TGF-β, mammalian target of rapamycin (mTOR) and hypoxia-inducible factor (HIF) signaling pathways, USP22 and USP21 help to maintain the fitness of Tregs within the TME. The simultaneous deletion of both USPs in Tregs led to an obvious decrease of Foxp3, altered Treg metabolic signatures, and impaired Treg-suppressive function, as well as alleviated the suppressive impact of Treg on cytotoxic CD8 + T cells [106] (Fig. 2D).

The development of specific USP22 inhibitors remains a great challenge. Based on these findings, researchers adopted the computer aided drug design (CADD) for developing a USP22-specific inhibitor with small molecular weight in vitro [93, 107]. Besides, Xu's group developed a nanopacked therapeutic system (galactose-decorated lipopolyplex, Gal-SLP) possessing self-activated cascade-responsive sorafenib and USP22 shRNA codelivery. Gal-SLPs have powerful anticancer effects via a trifold synergistic impact towards hepatocellular carcinoma [108]. However, the practical application of this research discovery is still limited due to biological safety concerns.

## USP14

### USP14 structure

The human USP14 protein consists of 494 amino acids and two unique domains. The N-terminal ubiquitin-like (UBL) domain controls proteasomal activity, and the C-terminal USP domain controls USP14's deubiquitinating enzymatic activity [109]. The catalytic domain of USP14 is composed of 3 subdomains, the finger, palm, and thumb, forming the ubiquitin-binding cleft. 2 surface loops within palm subdomain, BL1 and BL2, partially hover above the active site cleft and block the binding of the C-terminus of Ub [110]. The proximity of the two surface loops above the catalytic sites to the ubiquitin-binding groove prevents the C-terminus of Ub from binding to the active site of USP14, hence free USP14 exhibits lowered deubiquitinating activity [111]. However, after interaction with the 19S RP of the proteasome, USP14 goes through a major conformational shift, moving the two surface loops and permitting access of the ubiquitin C-terminus to the active site [112, 113]. Seven different phosphorylation sites, namely Thr52, Ser143, Ser230, Thr235, Ser237, Ser302 and Ser432, have been found on USP14. Ser143 and Ser432 are two of them that have been revealed to be phosphorylation sites for Akt [114, 115].

### Immunoregulatory function of USP14 in cancers

**Fig. 3:**
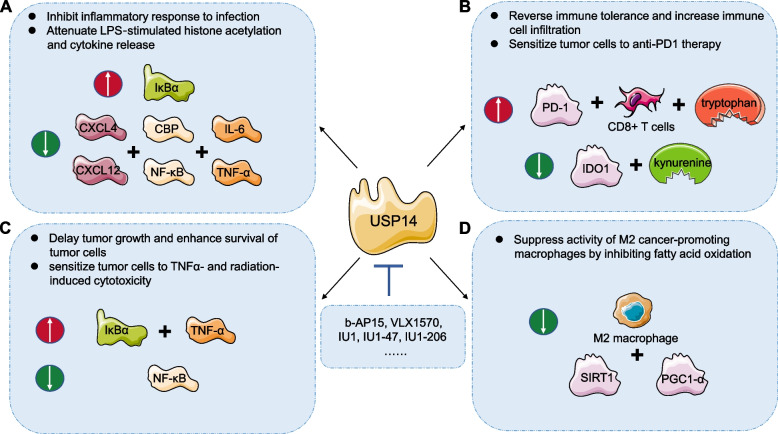
The effect by targeting inhibition of USP14 on anti-tumor immune response: **A** Inhibit inflammatory response to infection by suppressing CXCL12-mediated CXCL4 de-ubiquitination, reduce CBP abundance and attenuate lipopolysaccharide (LPS)-stimulated TNF‐α and IL‐6 release. **B** Reverse immune tolerance through decreasing IDO1 protein levels and kynurenine (KYN)/ tryptophan (TRP) ratio, and sensitize tumor cells to anti-PD1 therapy by upregulating PD-1 expression and increasing CD8 + T cells infiltration. **C**. Delay tumor growth, enhance survival of tumor cells, and sensitize tumor cells to TNFα-mediated cell death, as well as radiation-induced cell death by inhibiting NFκB activity and preventing IκBα degradation, which is a critical inhibitor of the canonical NFκB pathway. **D** Suppress M2-type macrophages polarization by inhibiting SIRT1/PGC1-a-mediated fatty acid oxidation in macrophages

Emerging evidence confirms that USP14 crucially impacts immune response through controlling the turnover of important signaling molecules linked to inflammatory pathways [116]. For instance, CXCR4 is a chemokine receptor that remarkably impacts the immune system. Knockdown of endogenous USP14 blocks CXCR4 deubiquitination and leading to downregulated chemotactic response to CXCL12 [117]. USP14 displays the significant effect to promote inflammation by critically enhancing NF-κB activation and cytokine release [118]. During osteoarthritis, inhibiting USP14 reduces cytokine release and increases the abundance of the NF-κB inhibitor, IκBα, to attenuate pathogenesis [119, 120]. Additionally, USP14 stabilizes CBP, a histone acetyltransferase controlling histone modification and cytokine-encoding gene expression. USP14 inhibition reduces CBP abundance and decreases lipopolysaccharide (LPS)-stimulated TNF‐α and IL‐6 release (Fig. 3A) [121]. USP14 also involves in promoting the peptide ubiquitination to function in primary MHC I antigen presentation [122]. However, USP14 promotes retinoic acid inducible gene 1 protein (RIG-I) deubiquitination at K63, which is important for inhibiting antiviral immune reaction. Inhibiting USP14 in turn results in RIG-I-triggered TNF-α and IL-6 production in mice with virus infection [123, 124]. The inconsistent findings from several labs may be the results of various experiment environments and different cell status, deserving more deep researches.

Although UPS14 significantly impacts the innate immune and inflammatory response, there is only limited research studying its impact on anti-tumor immune effect. Indoleamine 2,3-dioxygenase 1 (IDO1) crucially affects tryptophan (TRP) degradation and kynurenine (KYN) accumulation, which contributes to the immune evasion and immune tolerance to anti-PD-1or anti-CTLA-4 therapy [125, 126]. USP14 has been found to bind with IDO1 and deubiquitinating it, preventing its degradation and promoting the TRP metabolism and the immune suppression of CRC tumors. Inhibiting USP14 lowers the IDO1 protein level, enhances the CD8 + T cells infiltration, reverses immune tolerance, as well as makes CRC tumor cells more sensitive to the anti-PD-1 therapy (Fig. 3B) [127]. In head and neck squamous cell carcinoma (HNSCC) cells, USP14 inhibition weakened NF-κB activity. From mechanistic perspective, USP14 could bind to IκBα, the critical inhibitor of the NF-κB pathway, hence removing IκBα K48-ubiquitination, promoting TNFα-induced IκBα degradation and decreasing NF-κB expression (Fig. 3C) [128].

As we mentioned, macrophages are critical players in the regulation of TME, and their metabolic phenotype plays a crucial role in determining their pro- or anti-tumor activity [129]. USP14 activation is required for the stabilization of SIRT1 and PGC1-α, which is necessary for macrophage fatty acid oxidation activation, and promotes M2-type macrophages polarization. USP14 inhibition in tumor mice breaks the immune-suppressive action exhibited by tumor-promoting macrophages, meanwhile greatly reshaping the immune TME in GC (Fig. 3D) [130].

### The developments and applications of USP14 inhibitors in cancers

As DUBs feature strong conservative property, it is of large challenges to discover the effective and selective USP14 inhibitors. Despite the nearly 40 USP14 inhibitors reported to far, the majority are inefficient and multitargeted drugs [35]. For example, researchers have discovered inhibitors, like b-AP15 to covalent inhibition of both USP14 and UCHL5 to induce the cathepsin-dependent apoptosis by inhibiting the UPS system [131]. The cysteine residue in the catalytic triad of USP14 is Cys114, while in UCHL5, it is Cys90. b-AP15 specifically creates a covalent connection with these cysteine residues when binding to them, and it has a slightly higher affinity for USP14 than UCHL5 [131]. The β carbons in b-AP15 is critical action site and may act as Michael acceptor moieties that enable above cysteine residues in USP14 and UCHL5 covalent interaction [131]. In Morgan’s et al. research, b-AP15, could powerfully induce TNFα-induced NFκB activity, and stablize IκBα to keep it from degrading. HNSCC cells were made more susceptible to radiation- and TNFα-mediated cell death by b-AP15 in vitro and in tumor xenograft models (Fig. 3C) [128]. However, b-AP15 also affects non-USP14 targets, possibly resulting in toxicity. A derivative of b-AP15 called VLX1570 was developed for enhancing the drug-like qualities and specificity of b-AP15. Even so, a recent phase I trial ended considering substantial pulmonary toxicities despite positive pre-clinical evidence [132].

In 2010, Finley's team discovered IU1, the first highly specific inhibitor of USP14 by using a high-throughput ubiquitin-7-amido-4-methylcoumarin (Ub-AMC) hydrolysis experiment [112]. IU1 interacts with USP14 residues His426, Tyr436, and Tyr476 via van der Waals, and hydrophobic interactions [133]. Previous research has shown that IU1 inhibits cell growth and stimulates apoptosis in cervical cancer and BC, acting as an anticancer agent [134–136]. In the present study, IU1 was showed to significantly lower IDO1 protein levels, inhibit IDO1-induced immune suppression and TRP metabolism, and eliminate the "off-target" impact exerted by IDO1 inhibitors. IU1 plus anti-PD-1 co-treatment dramatically lowered the tumor weights as well as prolonged mice survival (Fig. 3B) [127]. Additionally, in tumor-bearing mice, IU1 inhibits USP14, which impairs the suppressive action of cancer-promoting macrophages and significantly alters the composition of the immune microenvironment (Fig. 3D) [130].

Moreover, applying medicinal chemistry, additional strong IU1 derivatives such as IU1-47, IU1-206, and IU1-248 were discovered [133]. The crystal structures of USP14′s catalytic motif in association with IU1, IU1-47, IU1-206, or IU1-248 revealed that the IU1 inhibitors bind to the thumb-palm cleft pocket within the catalytic core, rather than the direct catalytic site inhibition [137]. As they have high-resolution crystal structures, scientists have used structural bioinformatics tools for identifying possible allosteric inhibitors of USP14 by combining molecular docking, molecular dynamic simulation and other techniques [138].

## Other USPs in cancer immunity

In this part, we will focus on summarizing the research progress of several other critical USPs which participate in tumor immunity. However, due to the limitations of present research, we will not introduce their structure and inhibitor development in detail. We believe that in the future, more and more immunomodulatory functions and inhibitor applications of these USPs will be explored and developed.

### USP8

USP8 is a DUB with two unusual SH3-binding motifs (SH3BMs) surrounding a binding motif (14–3-3BM) [139]. Gads is a crucial T cell antigen receptor (TCR) downstream signaling adaptor, and its SH3 domain has a higher binding affinity to the amino-terminal SH3BM [140]. USP8 is required for the normal homeostasis of T cells and TCR activation by upregulating Foxo1-mediated IL-7R release. And inhibition of USP8 especially decrease suppressive effect of Treg cells, leading to inflamed colitis [141]. USP8 also is capable of directly deubiquitinating and stabilizing the type II TGF-β receptor TβRII which expressed in cell membrane and tumor-derived extracellular vesicles (TEVs). In addition, USP8 expression improves TβRII + circulating extracellular vesicles (crEVs)-mediated T cell exhaustion as well as improves the resistance to chemotherapy in aggressive breast tumors [142].

There is ongoing controversy regarding USP8's function in controlling PD-L1. On one hand, pancreatic cancer tissues presented obviously higher USP8 levels and USP8 promoted the deubiquitination of PD-L1 protein. Through the activation of cytotoxic T-cells, combining with a USP8 inhibitor and anti-PD-L1 therapy especiallly reduced the proliferation of pancreatic tumors and increased the antitumor immunogenicity [143]. However, on the other hand, in Xiong’s et.al study, USP8 inhibition yet strengthened the PD-L1 protein expression by increasing K63-linked PD-L1 ubiquitination mediated by TRAF6 to combat K48-linked ubiquitination. USP8 suppression activates NF-kB signaling, which in turn stimulates the innate immune response as well as the MHC-I expression [144]. Meanwhile, LncRNA SNHG12 was discovered to bind to HuR, evaluate PD-L1 and USP8 levels to help NSCLC escape the immune system. By reducing PD-L1 and USP8 expression, lncRNA SNHG12 silencing constrained tumor progress and elevated the proportion of CD8 + T cells in NSCLC [145]. All in all, these researches suggest a potential treatment approach that combines the use of a USP8 inhibitor and PD-1/PD-L1 inhibition to increase anti-tumor activity.

### USP15

Latest researches have demonstrated that USP15 is essential for controlling Toll-like receptor (TLR) signaling, NF-kB and RIG-I signaling for the creation of type I interferons and pro-inflammatory cytokines, also TGFβ and p53 signaling pathway to affect the immunological and inflammatory TME during tumor progression [38]. For instance, a functional screen revealed USP15 to be a component of a regulatory complex made up of the TGFβ- receptor (TβR-I) proteins SMAD7 and SMURF2 [146]. Through the PI3K/AKT pathway, TGFβ signaling can then increase the synthesis of USP15, which stabilizes p53 by deubiquitinating it [147]. In PDAC, a recent study found that USP15 was related to the TGFβ signaling pathway, and aberrant USP15 overexpression is highly related to a poor prognosis [148]. In addition, USP15 is necessary for the metastasis and inflammatory response in MDA-MB-231 BC cells induced by TGFβ-signaling [149].

USP15 has also become a crucial regulator in T cell activation. Particularly, USP15 promotes the degradation of NFATC2, which negatively regulates T cell activation. Inhibiting USP15 improves the T cell activation in vitro as well as boosts T cell responses to tumor burden and bacterial infection in vivo [150]. Furthermore, USP15 loss increases IFN-γ production in methylcholantrene (MCA)-induced fibrosarcomas mouse model, and this increased susceptibility is associated with an overabundance of Treg cells and suppressor cells in TME [151]. Moreover, TET2 DNA dioxygenase is monoubiquitylated at K1299 to promote its activity and USP15 is reported to remove the K1299-linked monoubiquitin then negatively regulates TET2 activity [152]. Ablation of USP15 increases TET2 binding to Cxcl9, Cxcl10, and Cxcl11 promoters, which trigger the production of IFN-γ chemokines and boosts tumor-infiltrating lymphocytes to improve the responsiveness to anti-PD-L1 treatment [152].

### USP9X

USP9X, also referred to as FAM (fat facet in mice), is an X-linked USP involved in embryos and neural development [153]. As a crucial part of the TGFβ signaling pathway, USP9X inhibits the activity exhibited by the SMAD4 transcription factor [154]. USP9X can critically regulate the TCR and BCR signaling pathway [155, 156]. For example, USP9X acquires the ability to deubiquitinate ZAP70, which is an important component for TCR signaling [156]. USP9X is also necessary for inducing the PKCβ kinase activity in B lymphocytes upon BCR-dependent activation [156]. Depletion of USP9X significantly decreased phospho-CARMA1 levels in B cells, which lowered the number of CARMA1/Bcl-10/MALT-1 (CBM) complexes and weakened the proximal Ag receptor pathway circuit and NF-κB production in B cells and T cells [157]. In human T cell lines, USP9X knockdown decreased the expression of NF-κB induced by TCR signaling, as well as weaken T helper cells differentiated into naive CD4 + T cells [157].

USP9X controls tumorous functions such as cell adhesion, cell polarity, cell death and inflammatory processes in cancer cells [158, 159]. USP9X is abnormally expressed in HNSCC, BC, melanoma, NSCLC, and other human malignancies [160–163]. In oral squamous cell carcinoma (OSCC), USP9X deubiquitinates PD-L1 as well as maintain its protein stable expression [164]. The findings give USP9X a theoretical foundation as an immunotherapeutic target [164]. Izrailit et al. previously reported that USP9X was capable of forming a multiple complex with pseudokinase tribbles homolog 3 (TRB3) which collectively activated the Notch signaling under conditions of cellular stress [165]. G9, a largely selective USP9X inhibitor, was revealed to inactivate Notch signaling, lower the proinflammatory cytokines interleukin-1 beta (IL-1β) and C–C motif chemokine ligand 2 (CCL2) in a mouse TNBC model [166]. These molecular alterations were accompanied by decreased tumor inflammation, increased in antitumor immune response, and suppressed tumor development for TNBC [166].

### USP18

USP18 is the major DUB protein that responsible for clearing interferon stimulated gene 15 (ISG15) from conjugated proteins [167]. Prior research found that programmed loss of USP18 enhances ISGylation, whereas its augmentation reduces cancer growth by contributing to inflammation happen [168]. Upon viral infection or in response to type I and type III IFNs, LPS, TNF-α, or genotoxic stress, the USP18 is rapidly and strongly upregulated after these inflammatory stimuli [169–172]. The effects on interferon signaling of USP18 also affects tumor progression [173]. Due to the USP18 deletion in cancer cells, chemotherapy, radiotherapy and IFN-α treatment induces more severe apoptosis and makes cancer cells more sensitive to those therapies [174, 175]. Similar outcomes are obtained when USP18 is silenced in glioblastoma cells, which suggests that USP18 inhibition causes cells to undergo apoptosis with robust activation of caspase-8 and caspase-3 through enhancing the IFN-I pathway [176]. In addition, due to knockdown of USP18, more T cell chemoattractant CXCL10 is generated in mammary epithelial cells, accompanied with creating a tumor-suppressive microenvironment by attracting CD4 + T cells [177].

However, according to research by Hong et al., increased USP18 expression in tumor cells would in turn inhibit carcinogenesis, whereas decreased USP18 promotes tumor growth and lowers immunosurveillance by decreasing the exogenous synthesis of IFN-γ and the survival of cytotoxic T lymphocytes (CTLs) in TME [178]. In human leiomyosarcoma cases, decreased USP18 expression shapes the IFN-γ hypersensitive environment, making vascular smooth muscle cell proliferation, resulting in a worse clinical outcome [179].

The mechanism underlying extra-nodal diffuse large B cell lymphoma (EN DLBCL) is not well explored, and its prognosis is frequently poor [180]. By using bioinformatic analysis, USP18 was the primary immunological gene in EN DLBCL due to co-expressed prognostic immune genes network [181]. USP18 was lowly expressed in EN DLBCL, under the involvement in DC-modulating immune responses [181]. More importantly, a recent study by Arimoto et al. proposed the regulatory effect of nuclear USP18 on cancer cell pyroptosis, which helped to understand the prospective application of inhibiting USP18 in cancer immunotherapy from new perspectives [182]. This study determined the mechanism by which targeting USP18 induces cancer pyroptosis through activating the production of a group of atypical IFN stimulated genes (ISGs) in addition to conventional ISGs [182]. These results firmly establish the significance of USP18 targeting as a possible cancer immunotherapy strategy.

## Conclusions

The development of immunotherapy is a potent cancer treatment strategy. However, such type of therapy does not benefit all cancer patients, despite its great potential. Recent research has revealed significant details regarding how USP members affects cancer immunotherapy. Here, we are the first summarize the comprehensive and detailed relationship between USPs and cancer immunity. USP7, USP22 and USP14 are the most widely studied members of USPs involved in immune process. We describe the information on their composition and structure, mode of action, the modulatory function in tumor immunity, interaction with the immune chemokines, as well as the development of inhibitors and potential clinical applications was listed in Table 1.

**Table 1:** Biological properties, action mechanisms and potential preclinical/clinical applications of USP inhibitors involved in cancer immunity

USPs	Inhibitors	Mode of action	Cancer types	Major target and action mechanism	Potential clinical significance	Reference
USP7	P5091	Covalent (USP7/ USP47) IC50 (USP7): 4.2 µM	Lung cancer	Delayed tumor growth, promoted tumor infiltration of M1 MΦs and IFN-γ + CD8 + T cells, activated the p38 MAPK pathway and increased the expression of PD-L1	High expression of USP7 in was negatively correlated with innate and adaptive immunity. Targeting USP7, in combination with immunotherapy, should be considered for lung cancer treatment	[59]
			Colon cancer	Decreased IL-10 and elevated IFN-γ and TNF-α in tumor tissues and serum, promoted the cytotoxic activity of CD4 + and CD8 + T cells and suppressed Treg cells	Contributed to anti-tumor immunity and may be a candidate for cancer immunotherapy	[22]
			Multiple myeloma	Decreased DNMT1 levels to activate DNA damage response and apoptosis, suppressed hypoxic TME	DNA damage inducer RRx-001 + P5091 combination enhanced anti-tumor immune response and overcome therapeutic resistance	[85]
			Urothelial bladder cancer	Induced degradation of CCDC6	Promoted bladder cancer cells sensitivity to PARP-inhibitor drugs. PARP-inhibitor drugs + P5091 + RRx-001 offering novel immunotherapeutic strategy	[86]
	Almac4	Non-covalent IC50 (USP7): 0.0015 µM	Gastric cancer	Downregulated PD-L1 expression, attenuated PD-L1/PD-1 interaction and sensitized cancer cells to T cell killing in vitro and in vivo	Served as an anti-proliferation agent as well as a novel therapeutic agent in PD-L1/PD-1 blockade to promote the immune response	[60]
	P217564	Covalent (USP7/ USP47) IC50 (USP7): 0.48 µM	Lung adenocarcinoma and mesothelioma	Downregulated Foxp3 and Tip60, impaired the suppressive function possessed by Tregs	Promoted the efficacy of adenovirus-based tumor vaccine and anti-PD-1 monoclonal antibody therapy	[57, 82]
	P22077	Covalent (USP7/ USP47) IC50 (USP7): 8.01 µM	Acute myeloid leukemia	Inhibited CHK1 expression to disrupt the replication fork reset in DNA replication process and made cells more adaptive to DNA damage	The cytarabine + USP7 inhibitor P22077 combination can work synergistically to promote anti-leukemic action to overcome chemoresistance	[88]
	GNE6776	Non-covalent IC50 (USP7): 1.3 µM	Triple-negative breast cancer	Suppressed ABCB1 protein expression	Induced apoptosis and suppressed metastasis in chemoresistant TNBC, and may enhanced specific immunotherapy of TNBC in future	[90]
	HBX19818	Covalent IC50 (USP7): 28.1 µM	Lung cancer	Reduced M2-associated marker CD206 expression but not affect the M1-associated marker CD86 expression	Promotes TAM polarization into proinflammatory M1 macrophages by specifically suppressing M2 macrophages	[59]
USP22	S02 compound	Unknown	Lung carcinoma and melanoma	Diminished TME-induced Foxp3 up-regulation, altered Treg metabolic signatures, impaired Treg-suppressive function, and alleviated Treg suppression on cytotoxic CD8 + T cells	Reduced Treg cell fitness and consequently enhanced antitumor immunity	[106]
USP14	IU-1	Non-covalent IC50 (USP14): 4-5 µM	Colorectal cancer	Decreased tryptophan metabolism and IDO1 expression, reversed suppression of cytotoxic T cells	Inhibited immune suppression and increased responsiveness to anti-PD-1	[127]
			Gastric cancer	Inhibited deubiquitination of SIRT1 in macrophage and fatty acid oxidation amplification	Disrupted the suppressive activity of cancer-promoting macrophages and effectively reshapes immune microenvironment	[130]
	b-AP15	Covalent (USP14/ UCHL5) IC50 (UCHL5): 2.1 µM	Head and neck squamous cell carcinoma	Reduced both basal and TNFα-inducible NFκB activity, NFκB-dependent gene expression and the nuclear translocation of the NFκB subunit RELA	Sensitized cancer cells to TNFα- and radiation-induced cytotoxicity	[128]
USP8	DUBs-IN-2	IC50 (USP8): 0.28 µM	Pancreatic cancer	Downregulated PD-L1 protein expression, activated cytotoxic T-cells and the anti-tumor immunity	Reduced tumor invasion and migration, enhanced anti-PD-L1 immunotherapeutic efficacy	[143]
			Colon adenocarcinoma	Increased PD-L1 protein abundance, triggered innate immune response and MHC-I expression through activating the NF-κB signaling	Combination with PD-1/PD-L1 blockade activated the infiltrated CD8 + T cells to suppress tumor growth and improves the survival benefit	[144]
USP9X	WP1130	Non-covalent (USP5/ USP9X/ USP14/ USP24/ UCHL5) IC50 (Bcr-Abl): 1.8 µM	Oral squamous cell carcinoma	Inhibited deubiquitination of PD-L1 and destabilized its expression	Promoted T-cell immune surveillance and blocked tumor cell growth	[164]
	EOAI3402143 (G9)	Covalent (USP5/ USP9X/ USP14/ USP24/ UCHL5) IC50: unknown	Triple-negative breast cancer	Deactivated Notch, reduced the production of the proinflammatory cytokines CCL2 and IL-1β	Promoted antitumor immune response, and suppressed tumor growth	[166]
USP25	AZ1	(USP25/ USP28) IC50 (USP25): 0.62 µM	Colorectal cancer	Attenuated Wnt and SOCS3–pSTAT3 signaling, and potentiated immune responses	Inhibited colonic tumorigenesis, and can as a druggable target for gastrointestinal infections and cancers	[183]

USP7 is an extremely profitable target because it regulates the stability of several substrates that participate in control of tumor immune processes. The research on USP7 inhibitors is most advanced, including P5091, HBX19818 and GNE-6776, etc. [61, 64, 74–81]. USP7 expression has a direct effect on the important p53/MDM2 axis, which regulates cell cycle and tumor cell programmed death [63]. The possible combination of immunological regulation and p53 restoration through USP7 inhibition is especially fascinating to minimize chemotherapy-induced damage in vivo and in vitro [65]. In addition, Treg cells regulation has been identified as a potential action mechanism for USP7 inhibitors applications in immunotherapy [55, 57]. TAMs also play a role in another immune evasion mechanism mediated by USP7 inhibitors. TAM polarization towards proinflammatory M1 macrophages is induced by USP7 inhibition, enhancing anti-tumor immune responses [59]. TAM variety and flexibility impede clinical use of treatment methods that target mononuclear phagocytes. USP7 inhibitors' complicated activity may aid in overcoming the hurdle among various immune cell subsets. Furthermore, combing USP7 inhibitors with other immune-modulatory agents or chemotherapy greatly improve DNA damage effect and help tumor cells to overcome therapeutic resistance [83, 84]. However, further clinical validation of these findings is required. The effectiveness of USP7 inhibitors hasn't been proven in a carefully chosen cancer patient population employing immunological and genetic biomarkers which can represent the complicated biological functions of USP7. The implement of clinical trials for USP7 inhibitors would be more appropriate for patients with immunosuppressive TME, MDM2 overexpression, and p53 mutations resistant.

The role of USP22 in the anti-tumor immune TME has been becoming an emerging hotspot, especially its complicated regulating action on different immune cells subsets. On one hand, USP22 can initially promotes the activation of immune cells at the earliest stage, including T cells, NK cells and B cells. Inhibiting USP22 can inhibit the activation and stability of these immune cells to stop their immune killing [95–98]. However, on the other hand, inhibiting USP22 in turn would increase proportions of NK cells and CD8 + T cells, and destroy the fitness of Tregs, making ‘cold’ tumors into ‘hot’ tumors, thereby converting tumor cells that are fully resistant to ICIs immunotherapy to a sensitive state [100]. Inhibiting USP22 would lead to less activation of immune cells to immunosuppressive microenvironment or more infiltration of immune cells to immune-sensitive status, this issue of deeply associated mechanisms needs to consistently be solved. What’s worse, there is currently no known small-molecule inhibitor of USP22 used in studies. As we described, the regulating role of USP22 in immunological function is complex and incomplete area. The inhibition of USP22 may have unforeseen adverse impacts as well as broad functional alterations. Therefore, it is extremely challenging to create small-molecule USP22 inhibitors that enhance the selectivity of their intended substrates.

USP14 has been shown to act a crucial part among various innate immune processes, such as viral infection and inflammatory response. It is noteworthy that USP14 regulates both canonical and noncanonical NF-κB signaling pathways, leading to the promotion of autophagy and cytokine release [118–121]. Furthermore, USP14 has been reported to enhance antiviral immune response by increasing the stability of CXCR4, releasing more IL-6 and TNF-α cytokines [117], and accelerating MHC-I antigen presentation [122]. However, recent studies have reported conflicting results, indicating that the USP14 inhibitor IU1 can trigger the expression of cytokine release, thereby enhancing antiviral immune response [123, 124]. Interestingly, the inhibition of USP14 was showed to upregulate TNF-α expression in tumor cells and sensitize them to TNF-α and radiation-induced cytotoxicity [128]. More importantly, USP14 directly interacts with fatty acid synthase (FASN) and affect metabolic process [184, 185]. This critical metabolic effect of USP14 has significant implications for future research. Studies have shown that inhibiting USP14 can regulate amino acid metabolism balance and fatty acid oxidation, leading to an evaluation in CD8 + T cells and a declined infiltration of promoting-cancer M2 macrophages [130]. Despite the promising findings on USP14's role in innate immunity and relatively mature inhibitor development, research linking USP14 to tumor immunity and the applicability of USP14 inhibitors in cancer immunotherapy is still limited. Therefore, we hope that existing studies on USP14's role in innate immunity can guide future research on metabolic immunity and cancer immunotherapy.

In addition, USP8, USP15, USP9X and USP18 are also being reported by a growing number of top research institutes for their vital immunomodulatory functions in cancer progression. Several other USPs have also been shown to participate in the immune process of different cancers, which are listed in Table 2 [183, 186–196]. Among these USP members, most of them are reported to be involved in regulating the critical PD1/PD-L1 signaling pathway and influence immunotherapy effect. USP7, USP22, USP8, USP18, USP9X and USP5 have been demonstrated directly bind with PD-L1 to induce its deubiquitination and stabilization [60, 100–102, 143, 164, 186]. Inhibiting these USP members sensitizes tumor cells to immunosurveillance and enhances anti-PD-L1/anti-PD-1 therapy efficacy. The typical USP inhibitors, such as P5091 and WP1130, have been reported to promote anti-PD-1/PD-L1 therapeutic efficacy through significantly inhibiting the deubiquitination of PD-L1 [60, 164]. However, such regulatory and therapeutic effects are not unique, and opposite results have been found. For example, inhibition of both USP7 and USP8 has been also reported to lead to upregulation of PD-L1 [59, 144]. Suppressing the expression of USP12 and USP48 increased the resistance to anti-PD-1 therapy and decreased the therapeutic efficacy of PD-1 inhibitors [189, 195]. We hypothesized that the reason why USP has such a complex regulatory effect on PD1/PD-L1 depends on different cancer types, different tumor cell states, and even different TME and immune cell infiltration conditions. In addition, PD-1/PD-L1 exhibits a variety of protein post-translational modifications (PTMs), including glycosylation, phosphorylation, palmitoylation, SUMOylation, and acetylation [197]. Does these USPs regulate PD-1/PD-L1 deubiquitination expression more significantly than other PTMs? Are there other USP members involved in PD-1/PD-L1 regulation? After inhibiting the specific expression of a certain USP, do other USPs also control the deubiquitination of PD-1/PD-L1 in cancer cells, and which USP is the most dominant regulatory role? These problems need further experimental research and mechanism exploration in the future. We believe that these explorations will provide new insights into the design of rational therapeutic strategies to modulate the PD-1/PD-L1 pathway by targeting associated USPs in cancer immunotherapy.

**Table 2:** Other USPs participate in cancer immunity

USPs	Cancer Type	Immunoregulatory Mechanism	Reference
USP8	Breast cancer	Encourage tumor cell invasion, metastasis, and TGF-β/SMAD-induced epithelial-mesenchymal transition; Promote T cell exhaustion and create resistance to chemotherapy by deubiquitinating the TGF-β receptor TβRII	[142]
	Pancreatic cancer	Overexpressed in cancer tissues, promote tumor invasion and migration and tumor size; Interact positively with PD-L1, upregulate PD-L1 expression and decrease CD8 + T cells infiltration	[143]
	Lung cancer, Colon cancer, Melanoma	Inhibiting USP8 increases the PD-L1 expression through elevating the TRAF6-mediated K63-linked ubiquitination to antagonize K48-linked ubiquitination and degradation of PD-L1	[144]
	Non-small cell lung cancer	Silencing lncRNA SNHG12 restricts tumor growth and upregulated the ratio of CD8 + T cells by decreasing USP8 and PD-L1, inhibiting immune escape	[145]
USP15	Pancreatic ductal adenocarcinoma	USP15 and TGF-β are positively correlated and associated with poor prognosis	[147]
	Triple negative breast cancer	USP15 is required for metastasis triggered by TGF-β signaling	[148]
	MCA-induced fibrosarcoma Melanoma	T cell intrinsic USP15 deficiency causes excessive production of IFN-y, which increases Treg cells and CD11b + /Gr1 + myeloid-derived suppressor cells into immunosuppressive tumor microenvironment Suppress tumor immunity via deubiquitylation and inactivation of TET2, leading to decreased response to immunotherapy	[151, 152]
USP9X	Oral squamous cell carcinoma	Combined with PD-L1 to induce its deubiquitination and maintain stable expression to promote tumor cell growth and immune escape	[164]
	Triple negative breast cancer	Activate Notch signaling and promote CCL2 and IL-1β cytokine release to promote tumor inflammation and antitumor immune response	[166]
USP18	Breast cancer	Absence of USP18 leads to an increase in the induction of apoptosis by chemotherapy and treatment with IFN-α	[174]
	Chronic myeloid leukemia and colorectal carcinoma	USP18 regulates major histocompatibility complex class I (MHC-I) antigen presentation, PD-L1 expression and stimulation of a T cell response to enhance tumor cell antigenicity and radiosensitivity	[175]
	Glioblastoma	Silence USP18 to enhance IFN-I pathway causes drug-treated cells to undergo apoptosis with robust caspase-8 and caspase-3 activation	[176]
	Melanoma	Regulate IFN-γ-mediated immunoediting, including upregulating MHC class-I expression, reducing tumor cell-mediated inhibition of T cell proliferation and activation, and suppressing PD-1 expression in CD4 + and CD8 + T cells to sensitize tumor cells to immunosurveillance and immunotherapy	[178]
	Extra-nodal diffuse large B cell lymphoma	Downregulation of USP18 was associated with reduced aDC number in the tumor tissues	[181]
	Acute myelocytic leukemia	USP18 suppression not only enhances expression of canonical IFN stimulated genes (ISGs), but also activates the expression of a set of atypical ISGs and NF-κB target genes to induce cancer pyroptosis	[182]
USP5	Non-small cell lung cancer	USP5 directly interacted with PD-L1 and deubiquitinated PD-L1 to promote cancer progression	[186]
USP6	Ewing Sarcoma	Enhance chemokine production in response to exogenous IFN by inducing surface upregulation of IFNAR1 and IFNGR1, stimulate migration of primary human monocytes and T lymphocytes and triggered activation of natural killer (NK) cells	[187]
USP10	Pancreatic adenocarcinoma	Inhibit YAP1 ubiquitination and degradation to promote Cyr61 expression, induces immune escape and promotes growth, metastasis and trigger M2 polarization of macrophages	[188]
USP12	Lung adenocarcinoma	Downregulation of USP12 promotes an immunosuppressive microenvironment with increased macrophage recruitment, hypervascularization, reduced T cell activation and enhances resistance to anti-PD-1 immunotherapy	[189]
	Colon adenocarcinoma	Positively regulates myeloid-derived suppressor cells (MDSCs) function and PD-L1 expression to inhibit antitumor immunity, through deubiquitinating and stabilizing p65	[190]
USP20	Breast cancer	TINCR upregulate USP20 and PD-L1 through the dual role of ceRNA interaction and miR-199a-5p transcription inhibition, to induce immune escape and disease progression	[191]
USP25	Colorectal cancer	Supports colonic inflammation and bacterial infection and promotes colorectal cancer	[183]
USP35	Skin Cutaneous Melanoma	USP35 overexpression is associated with an unfavorable prognosis and an immunosuppressive TME, by downregulating the abundance of infiltrating CD8 + T cells	[192]
USP43	Pancreatic ductal adenocarcinoma	Overexpression of USP43 is a potential prognostic indicator for PDAC patients and associated with T cell activation, suppression of CD8 + T cell enrichment, and the cytokine signal pathway	[193]
USP44	Colon adenocarcinoma, melanoma and lymphoma	Promotes FOXP3 stabilization and Treg function during inflammation to inhibit antitumor immunity	[194]
USP48	Pancreatic adenocarcinoma	Enhances the therapeutic efficacy of PD-1 inhibitors, promotes functions of T cells and tumor-associated macrophages, and induces pyroptosis	[195]
USP51	Non-small cell lung cancer	USP51/PD-L1/ITGB1 axis potentially contributes to the malignant progression and chemoresistance	[196]

In this review, the study of USP deubiquitinases in cancer immunotherapy has shown promising results, with several basic and preclinical researches demonstrating their potential as immunotherapeutic targets. However, there are still some limitations to consider. The role of USPs in regulating immune responses is complex and context-dependent, and their effects and detailed mechanisms on tumor cells versus immune cells need to be further elucidated. Additional and more importantly, the development and specificity of USP inhibitors is still a great challenge need to be carefully evaluated and explored. One of the challenges is the potential for off-target effects. Because USPs are involved in a variety of biological processes, inhibition of USPs could potentially impact multiple pathways and lead to off-target effects. Another challenge is current mostly USP inhibitors lack of accurate targeting specificity. Moreover, the heterogeneity of tumors may also limit the efficacy of targeting USPs, as different tumors may have different mechanisms of immune evasion. Targeting a single USP may not be sufficient to overcome these mechanisms and the compensatory mechanisms possibly restrict the efficacy of targeting USPs alone.

Therefore, to advance the development of USP inhibitors, there is still a need for more precise mechanisms of interaction between small molecules and USPs and more accurate screening methods. One strategy is to perform high-throughput screening assays for identifying compounds selectively inhibiting specific USP family members associated with cancer immunity. These assays can be carried out in vitro or in vivo, using cell lines or animal models that mimic TME. Another approach is to use computational biology and structure-based drug design for confirming compounds that exhibit stronger affinity and specificity for the active sites of specific USPs. Integrating genomic and transcriptomic analysis of tumors and immune cells can also be applied to provide insights into the expression and activity of specific USPs in different tumor types and immune cell subsets, which can guide the selection of the most effective USP inhibitors. In addition, exploring potential biomarkers can help identify patients who are most likely to benefit from USP inhibitor therapy. Researches can investigate the use of biomarkers, such as gene expression signatures or protein markers, to predict response to USP inhibitors. Furthermore, more preclinical study may focus on exploring combination therapies that target USPs in combination with other therapies, such as ICI or chemotherapy. This approach can try to avoid the problem of USP inhibitor resistance from the beginning and enhance the therapeutic efficacy. Finally, clinical trials can provide insights into the efficacy of USP inhibitors in specific patient populations to solve the issue of complex and compensatory mechanisms in different tumor types as much as possible.

In conclusion, finding synergistic USP inhibition mechanisms and creating combinatorial therapeutic strategies for cancer immunotherapy should be the main goals of future research. By combining multiple approaches, we believe that can identify the most effective USP inhibitors for different types of tumors and strongly assist in developing personalized cancer immunotherapies in the future.

## Data Availability

Not applicable.

## Abbreviations

MHC: Major histocompatibility complex
Treg: Regulatory T cells
MDSC: Myeloid-derived suppressor cell
NK: Natural killer
DC: Dendritic cell
ICI: Immune checkpoint inhibitor
PD-L1: Programmed Cell Death 1 Ligand 1
NSCLC: Non-small cell lung cancer
RCC: Renal cell carcinoma
UPS: Ubiquitin–proteasome system
DUB: Deubiquitinating enzyme
USP: Ubiquitin-specific protease
Ub: Ubiquitin
TRAF: Tumor necrosis factor Receptor-Associated Factor
ICP0: Infected Cell Polypeptide 0
DNMT1: DNA methyltransferase-1
MDM2: Mouse double minute 2
UHRF1: Ubiquitin-like with PHD and Ring Finger Domains 1
Foxp3: Forkhead box protein P3
Tip60: Tat-Interactive protein
CTLA4: Cytotoxic T-lymphocyte-associated protein 4
TAM: Tumor-associated macrophage
siRNA: Small interfering ribonucleic acid
MAPK: Mitogen-activated protein kinase
CHK1: Checkpoint kinase 1
BC: Breast cancer
CRC: Colorectal cancer
TNBC: Triple-negative breast cancer
Ubp8: Ubiquitin carboxyl-terminal hydrolase 8
ZnF UBP: Zinc-finger ubiquitin-binding domain
BMI1: B cell-specific Moloney murine leukemia virus integration site 1
FBP1: Far-upstream element–binding protein 1
SIRT1: Sirtuin 1
PDAC: Pancreatic ductal adenocarcinoma
TGF-β: Transforming growth factor–β
IDO1: Indoleamine 2,3-dioxygenase 1
TRP: Tryptophan
KYN: Kynurenine
HNSCC: Head and neck squamous cell carcinoma
GC: Gastric cancer
SH3BM: SH3-binding motif
TCR: T cell antigen receptor
TEV: Tumor-derived extracellular vesicle
crEV: Circulating extracellular vesicle
PBMC: Peripheral blood mononuclear cell
CBM: CARMA1/Bcl-10/MALT-1
OSCC: Oral squamous cell carcinoma
CCL2: C–C motif chemokine ligand 2
IL-1β: Interleukin-1 beta
ISG15: Interferon stimulated gene 15
CTL: Cytotoxic T lymphocyte
EN DLBCL: Extranodal diffuse large B cell lymphoma
